# Influence of preventive sex education programmes in compulsory secondary education students: a descriptive observational study

**DOI:** 10.1186/s12889-022-14649-w

**Published:** 2022-11-24

**Authors:** Arturo Hidalgo Berutich, María Barbosa Cortes, Esther Cárdenas Feria, Margarita Carrillo Rufete, Miguel Pedregal González, Eduardo Perez Razquin, Esteban Delgado Arcos

**Affiliations:** 1 Clinical Management Unit “Andevalo Occidental”, Huelva-Costa Primary Healthcare District, Andalusian Health Service, Andalusia, Spain; 2 Clinical Management Unit “Torrejon”, Huelva-Costa Primary Healthcare District, Andalusian Health Service, Andalusia, Spain; 3 Obligatory Secondary Education Teacher, Pedagogue, Counsellor at “IES del Andevalo” Secondary School, Department of Education, Regional Government of Andalusia, Andalusia, Spain

**Keywords:** Adolescent, Sexual behaviour, Sexual education, Forma Joven

## Abstract

**Background:**

Sex education programmes conducted by health professionals and educators are essential for young people to adopt healthy habits and attitudes towards their sexuality. The Forma Joven Program, promoted by the Andalusian Regional Government’s Ministry of Health and Families and Education, is a good example of this. The aim of the study is to determine if different “degrees of intervention” in the informative consultancies of the Forma Joven Program imply differences in knowledge and attitudes towards sexuality.

**Methods:**

This descriptive observational study analysed 27 Compulsory Secondary Education high schools in Huelva. These were included in the Program and belonged to a Primary Care Health District. Out of the 17 institutes initially selected because they met the inclusion criteria (4.256 students), finally 14 (3.596 students) participated. During the 2018/2019 school year, students from 3rd, 4th year of Secondary Compulsory Education (ESO), 1st, and 2nd Baccalaureate of the selected centres were asked to fill in a questionnaire of knowledge and attitudes towards sexuality. It collected variables such as age, sex, school year, institute, educational level and employment status of the father or mother and profile of the professional who delivers the counselling.

**Results:**

A total of 1.237 students completed the questionnaire, which represents a participation rate of 34.4%. The average age was 15.59 years (SD 1.26) and 39.9% were girls. In some evaluated questions, we found statistically significant differences between the groups with different levels of exposure to counselling and the acquisition of knowledge and attitudes towards students’ sexuality, although in most of them no such differences were found. The results of this study suggest the importance of the quality of counselling over quantity. Some classic myths persist in relation to sexuality and in some situations, they can be decisive when adopting preventive measures to avoid risks related to pregnancy and contagion of STIs.

**Conclusions:**

A greater number of counselling sessions does not imply acquiring a higher level of knowledge or better attitudes towards sexuality. Perhaps the quality of the education is more important than the quantity of counselling sessions.

**Supplementary Information:**

The online version contains supplementary material available at 10.1186/s12889-022-14649-w.

## Background

Primary care health professionals should integrate sex education into preventive programmes, complementing the information adolescents receive from their families, peer groups and schools [1]. An issue of great relevance and topicality in reference to sex education is the influence of the Internet and social networks on it. It is not in vain that it is the source from which adolescents obtain most of their information [2].

It is advisable to provide clear and coherent messages about contraceptive methods during adolescence. It is important to provide accurate information and to emphasise the need for shared responsibility in sexual relations [3]. It is also essential to know that the theoretical efficacy of a method can be far from the real one if it is not used in a continuous and proper way [4, 5]. 20% of Andalusian adolescents between 11 and 17 years old who have had coital sexual relations did not use condoms, and 5.2% did not use any method. 2.5% of girls had had at least one pregnancy, with the highest risk age being 15–16 years old. Regarding voluntary termination of pregnancy, there is an upward trend in Andalusia [6].

Concerning sexually transmitted infections (STIs), since 2011 there has been an increase in Andalusia due to a lower perception of risk [7], with the consequent lower use of preventive measures such as condoms, as well as the emergence of new sexual behaviour patterns. The age of initiation of sexual relations has advanced in recent years. In Andalusia, the average age for coital sexual debut is 15 years old [8].

A study [9] by the Youth and Social Inclusion Network and the University of the Balearic Islands on pornography consumption among young people has recently been published. This study reveals that one in four boys watch pornography before the age of 13 and the first access to pornography is as early as 8 years old. There has been a progressive increase in pornography consumption among girls over the last 5 years. 33% of the boys who watch pornography report that they do so to learn about sex. The easy access to new technologies through mobile screens makes the contents of the “new pornography” the main source of information on sex education for adolescents.

We cannot ignore the fact that the messages contained in this type of pornography have a strong sexist component and encourage violence and risky practices: repetitive ritual practices, risky behaviour (non-use of barrier methods), violent relationships (it is not uncommon to see videos in which women are subdued against their will). Moreover, the type of interpersonal relationships they engage in are based on sporadic contact with multiple people at the same time and without a previous relationship. It is not so much a question of “banning” the consumption of pornography but rather of critically analysing what is presented on it. The authors conclude that the only possible way to try to counteract the enormous influence of the new pornography on our young people must be based on sex education programmes in schools and aimed at parents, for them to provide adolescents with the tools to make decisions when it comes to living their sexuality.

In this regard, educational programmes in schools and educational institutions have been shown to be effective in providing information to adolescents and parents on STI prevention, HIV, and contraception [10–22]. There are few studies demonstrating the effectiveness of internet-based educational interventions for this purpose, although it should be an increasingly used resource [23].

All these aspects are addressed in the Forma Joven Programme.

Forma Joven is an inter and multidisciplinary Health Program that has been developed in Andalusia since 2001 and in which professionals from various sectors related to the health and education of the adolescent population participate [24]. The Program was born as a need to respond to the educational and informative aspects that this group systematically raises, and tries to intervene in “risk situations” that derive from certain attitudes and behaviors to try to modify and reduce their negative effects on the health from promotion and prevention (Diezma & De la Cruz, 2002; Hernández Gutiérrez et al., 2000).

It covers five basic areas:

1. Socio-emotional education.

2. Healthy lifestyles.

3. Sexuality and egalitarian relationships.

4. Positive use of Information and Communication Technologies (ICTs).

5. Prevention of consumption of alcohol, cannabis and other drugs.

The activities carried out in the Program to address said contents are:

Informative consultancies, Workshops, Group talks and Training of mediators.

The Forma Joven strategy was born with the aim of approaching those spaces that young people and adolescents frequent: both educational centers and other youth meeting spaces. It is in the educational field where Forma Joven has had a greater evolution and permanence in relation to the affiliation of centers and actions aimed at promoting healthy environments and behaviors among young people.

Informative advice has become a privileged and permanent “observatory” that is essential to meet the real information needs of adolescents and to introduce appropriate modifications both in the type of interventions and in their content. It is a space where students come voluntarily individually or in small groups (2–6) to raise their doubts directly with the professional who attends to them. During the development of the consultancies, an approach is attempted from the philosophy of “meaningful learning” and the gender perspective, where they themselves are actors in the construction of their own knowledge. Although, as mentioned above, the program includes a wide variety of interventions developed by the professionals involved in it (workshops, meetings, group talks, training of health agents), counseling can be considered the “main core” of the program. Since this is where the boys and girls raise their “real” doubts about the issues that concern them. In addition, we consider that the information provided to them during their attendance at them will probably be extended to the rest of the peer group due to the “wave effect” that is generated among them. In this way, a “potentiation” of the effects of our interventions is produced. It can be said that it is a program adapted to the adolescent population of each educational center, and based on their demands and needs for information and training.

Informational advisories are held during school hours, typically 1 hour per week. The students of the center can go to these advisory services on their own initiative and by requesting an appointment with the counselor. They take place in a physical space located in the center (preferably the counselor’s office), specifically adapted to the situation where, in addition to preserving privacy and confidentiality, graphic support material is available for the sessions and internet connection. They will be attended by the counselor and/or a health professional from the health centers who usually come to the educational center at that time. This is the fundamental activity of the program and the basis for guiding the contents to be developed in the rest of the activities. For this reason, it has been decided to select the criterion of “level of exposure to consultancies” to compare the different groups.

The group sessions and workshops are carried out both by collaborators outside the educational center (doctors, nurses and social workers), as well as by the center’s own teachers (teachers and counselor). Their contents must be adapted to each group. The coordination of the activities corresponds to the counselor or person in charge of the program in the educational center.

The mediators are male and female students of the school who, due to their specific characteristics (leadership, learning capacity and knowledge transmission), are selected by the center’s teachers and counselor to interact with their peers and act as educators among their peers.

According to the latest data published by the Forma Joven programme, professionals have covered 821 points located in educational centres in Andalusia. A total of 300.966 adolescents (153.666 boys and 114.7297 girls) have been assisted in counselling sessions [25, 26].

The counselling sessions have become a privileged and permanent “observatory” essential to know the real information needs of adolescents and to introduce modifications in the educational interventions implemented in educational centres. During the counselling sessions, the approach is based on the philosophy of meaningful learning and gender perspective, where adolescents are the main actors of their own knowledge construction.

Counselling can be considered the core of the Programme, since it is in these sessions where the boys and girls raise their real doubts about the issues that concern them. They take place during school hours, generally 1 hour a week. Students can attend individually or in small groups (2–6). They take place in a physical space located in the centre, specifically adapted to the situation, preserving privacy and confidentiality. This is the key activity of the programme and the basis to guide the contents to be developed in the rest of the activities. For this reason, it was decided to select the criterion of “level of exposure to counselling” to compare the different groups.

In terms of the literature on the subject, there are different studies published on sexuality and contraception in adolescence. However, no article has been found on the evaluation of the results of sex education interventions in a collaborative environment between educators and health professionals. We did find a recent publication that evaluates the Forma Joven Programme in three areas: sexuality, consumption of addictive substances and road safety.

This is a quasi-experimental study conducted in 50 secondary schools in the city of Seville that evaluates the impact of the Forma Joven Programme, comparing the participating students with a control group. It concludes that there are no significant differences between the two groups, and that there is a lack of systematisation in the implementation of the programme’s records. In contrast to this study, significant differences were found in some questions related to attitudes towards sexuality in adolescents in an educational intervention conducted in two compulsory secondary schools (intervention group/control group) in Huelva, with a sample of 222 students [27].

In a Cochrane systematic literature review on the effectiveness of school-based adolescent sexuality education programmes in reducing STIs and unintended pregnancy, the authors conclude that there is little evidence that curriculum-based education programmes alone are effective in improving adolescent sexual and reproductive health outcomes.

Incentive-based interventions focused on keeping young people in secondary school may reduce adolescent pregnancy, but we need more trials to confirm this [28].

For this reason, we decided to carry out this project in the adolescent population of the secondary schools in the so-called Huelva-Costa Health District in the province of Huelva, to study whether different “degrees of intervention”, determined by the number of counselling sessions conducted lead to differences in the knowledge and attitudes of the adolescents, as well as to determine whether the number of counselling sessions is related to certain characteristics of the teacher. This is a novel research project, as there are no publications in this area in the province of Huelva. Initial hypothesis: “A greater number of educational interventions on knowledge and attitudes towards sexuality in compulsory secondary school students generate an increase in said knowledge and an improvement in attitudes”.

As reflected in the Madrid consensus document “Science-based sexuality education” [29], an international group of experts on the subject proposes to conduct surveys on the attitudes towards sexuality of children and adolescents, as a strategy to design the educational curricula. The Forma Joven programme meets many of the characteristics suggested in that document as “important” for effective sexuality education programmes.

The main objective of this study is to evaluate whether different levels of educational intervention on sexuality, quantified by the number of informative counselling sessions, lead to differences in the knowledge and attitudes of adolescents participating in the Forma Joven Programme.

Other purposes are to analyse knowledge and attitudes towards sexuality and the use of contraceptive methods in students in Compulsory Secondary Education and Baccalaureate, and determine whether there are differences between groups according to the different levels of educational intervention, based on the activity log of the official platform Forma Joven from the Ministry of Health and Families of the Andalusian Regional Government, specifically on the number of counselling sessions conducted in the centres.

## Methods

### Study design

This is a descriptive observational study in which informative counselling was selected as the educational intervention for the groups.

By consensus of the research team, we formed three groups: schools that are above the 90th percentile in the number of counselling sessions during at least two of the last 4 years (exposure level 1), centres that are between the 50th and the 90th percentile in the number of counselling sessions during at least two of the last 4 years (exposure level 2) and schools that are below the 50th percentile in the number of counselling sessions during at least two of the last 4 years (exposure level 3).

### Participants

**Fig. 1:**
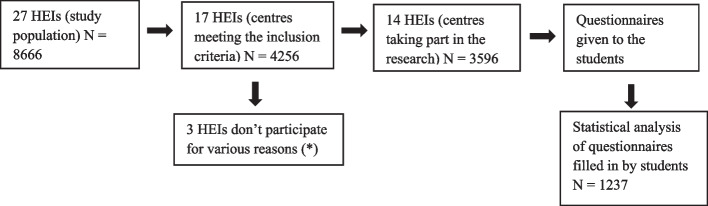
Study stages (*) Possible conflict situations with parents over sexuality issues”, “not being a priority issue for the school”, “work overload for teachers”, “tutors not having enough time in their schedules”, “teachers’ workload”

The study population is made up of students in the 3rd, 4th year of Compulsory Secondary Education (ESO), 1st and 2nd year of Baccalaureate from 27 Higher Education Institutes (HEIs). A total of 8.666 students. The sample coincides with the study population as all students will be included (Fig. 1).

### Procedures

Data collection was performed via the “Questionnaire of attitudes and knowledge towards sexuality” (Appendix 2) given to the students in the selected schools. It was self-administered and anonymous.

This questionnaire was based on the questionnaire developed by Barella Balboa JL and collaborators [30] with the consent of the authors. It was modified and adapted to the study population by adding other relevant questions. To this end, the questionnaire was validated by means of a judge’s test with four members of the research team (informed opinion of people with experience in the subject and acknowledged as qualified experts) and piloted on 104 students.

The questionnaire consists of 26 questions with four possible answers following a Likert scale (Cronbach’s alpha of 0.629).

The questionnaire was administered during the 2018/19 school year to all students attending the schools included in the study and where the Forma Joven programme is already being implemented. From the originally included schools, we selected the ones meeting the inclusion criteria. As inclusion criteria, we chose the schools belonging to the Forma Joven Programme within the Huelva-Costa Health District (geographical area) which have been participating uninterruptedly for the last 4 years, in which at least informative counselling sessions are conducted and which agree to participate in the study (signing a written informed consent form).

The variables collected were age, sex, school year (3rd, 4th ESO, 1st, 2nd Baccalaureate), secondary school where the child is studying, educational level of the father and mother, employment status of the father and mother, and profile of the professional providing counselling.

The sections father’s and mother’s educational level and employment situation were measured according to the variables shown in Appendix 1. Data collection on the profile of the counsellors was carried out through a questionnaire (Appendix 3).

### Statistical analysis

A descriptive analysis of the included variables and the comparison of both groups was performed. Continuous data is presented using mean and standard deviation. Categorical data is given as frequencies with percentages. A bivariate analysis was used to establish the possible differences between the groups. In this bivariate analysis, a proportion contrast test based on the Chi-square test (χ2) was used to test the relationship of dependence between categorical variables.

The one-way analysis of variance (ANOVA) was used to compare quantitative variables. Normal distribution will be measured by using Komogorv-Smirnov test. For each analysis a probability value of *p* < 0.05 will be considered statistically significant. Data were analysed using the Statistical Package for Social Sciences (SPSS) program (V. 24.0, SPSS Inc., Chicago, IL, USA).

### Outcomes

Seventeen schools were selected out of the 27 schools initially included (*N* = 8.666) as meeting the inclusion criteria (*N* = 4.256). Fourteen of these schools participated in the study (*N* = 3.596), as three of them refused to be included by their management for various reasons (possible conflict situations with parents due to sexuality issues, not being a priority issue for the school, work overload for teachers, lack of time available for tutors in their schedules). This represents a participation rate of 82.35% of the centres. Finally, the total number of questionnaires completed by students was 1.237. The difference between the number of students initially selected to fill in the questionnaire (3.596) and the number who finally answered it (1.237) is due to two fundamental reasons: the absence of a certain number of students on the day the intervention was carried out and non-presentation of the mandatory consent to participate in the study, by the parents. In this sense, it is possible that the motivation and awareness of parents towards sexual education may be an important factor to take into account in the self-selection of the respondents.

Regarding the lower number of girls who answered the questionnaire, we did not find any explanation. Girls accounted for 39.9% and the average age of the sample was 15.59 (1.26) years.

The data obtained from the analysis of the profile of the professionals who carry out information counselling on sexuality are shown in Table 1.

**Table 1:** Socio-demographic descriptors of students and professionals

		% Average (dt) (*N* = 1.237)
Gender (female)		39.9%
Age		15.59 (1.26)
Academic Year	3rd Compulsory Secondary Education	42%
	4th Compulsory Secondary Education	34.3%
	1° Baccalaureate	12.9%
	2° Baccalaureate	10.8%
Exposure to consultancies	> p90	23.4%
	p50–90	35.9%
	<p50	40.7%
Consulting professional	Doctor	23.1%
	Nurse	7.7%
	Social Worker	38.5%
	Teachers	7,7%
	Guidance counsellor	23.1%
	Others	0,0%
Gender (male)		53.8%
Professional age		48,42 (7,81)
Previous training		92,3%
Years providing consultancy services		

According to the responses, the majority answered that their main source of information on sexuality is friends (74.6%), followed by talks/workshops (41.7%) and the Internet (35.9%).

### Relationship between the level of exposure and the survey questions

In terms of “exposure level” to counselling, 23.4% are above the 90th percentile, 35.9% are between the 50th and 90th percentile and 40.7% are below the 50th percentile. Regarding the differences in the variables according to the three levels of exposure to consultancy, we found statistically significant differences. The most relevant results are shown in Table 2.

**Table 2:** Relationship between the level of exposure and the survey questions

Question	Percentile	Strongly agree	Somewhat agree	Somewhat disagree	Strongly disagree	p
5. The most important thing in sexual intercourse is penetration.	**>p90**	**7,8%**	**23,7%**	37,8%	30,7%	0,023
	p50–90	6,4%	16,2%	38,5%	39,0%	
	<p50	6,9%	16,3%	34,5%	42,3%	
10. If a girl has penetrative intercourse and does not orgasm, she cannot become pregnant.	**>p90**	**8,6%**	**7,1%**	11,4%	72,9%	0,024
	p50–90	3,0%	4,8%	12,6%	79,6%	
	<p50	5,7%	5,7%	9,7%	78,9%	
19. If you have penetrative sex without using a condom during menstruation, there is no danger of pregnancy.	**>p90**	9,4%	16,9%	**28,4%**	**45,3%**	0,012
	p50–90	7,8%	16,5%	24,5%	51,1%	
	<p50	7,7%	12,7%	20,2%	59,5%	
20. The first time a girl has penetrative intercourse it always hurts and bleeds.	>p90	26,5%	36,7%	24,0%	12,7%	< 0,001
	p50–90	18,7%	34,0%	27,6%	19,6%	
	**<p50**	12,2%	30,3%	**34,3%**	**23,1%**	
24. Jealousy is normal when a boy and a girl are in love.	>p90	12,4%	24,7%	30,7%	32,2%	0,031
	p50–90	13,1%	23,0%	23,4%	40,5%	
	**<p50**	13,7%	18,1%	**25,3%**	**42,8%**	
25. To have a good relationship, the girl should avoid contradicting the boy.	**>p90**	2,1%	0,7%	**9,9%**	**87,3%**	0,04
	**p50–90**	2,3%	1,6%	**10,4%**	**85,7%**	
	<p50	3,6%	3,8%	7,8%	84,8%	

Statistically significant differences were found in questions 5 and 10 (*p* = 0.024; *p* = 0.023, respectively), with the percentile> 90th exposure group having a higher percentage of “Strongly agree” and “Somewhat agree” responses. In question 19 there were also statistically significant differences (*p* = 0.012), with a higher percentage of “Somewhat disagree” and “Strongly disagree” responses in the > 90th percentile group.

In questions 20, 24 there were statistically significant differences (*p* < 0.001; *p* = 0.031), with the percentile group < 50 having a higher percentage of “Somewhat disagree” and “Strongly disagree” responses. In question 25 there were statistically significant differences (*p* = 0.040), with the percentile group>90 and 50–90 having a higher percentage of “Somewhat disagree” and “Strongly disagree” responses.

Statistically significant differences were found with respect to the relationship between fathers’ educational and occupational level (*p* = 0.006, *p* = 0.008) and mothers’ educational and occupational level (*p* = 0.083, *p* = 0.033) with the exposure level to counselling.

However, with respect to the statistical analysis carried out to test the relationship between the number of counselling sessions held in each centre and the profile of the teacher, and between the level of studies and occupation of fathers/mothers and the different institutes, it was found that the conditions for the application of the Chi-square test were not met.

A multivariate analysis was not performed since no significant differences were found in the results when comparing the different degrees of exposure with the answers to the questions in the questionnaire.

## Discussion

This is the first study to relate the number of informative counselling sessions to the level of knowledge and attitudes towards sexuality of adolescents participating in the Forma Joven programme in Andalusia.

The findings show that more counselling is associated with a higher level of knowledge and better attitudes of young people towards sexuality in some areas assessed in the questionnaire, but not in many others.

The results may seem paradoxical, but the improved level of knowledge and attitudes probably depends more on the quality of the counselling than on the quantity of counselling sessions.

The freedom of implementation of the Programme and the different training levels of the professionals involved, imply that there are no homogeneous criteria or protocols to conduct the different activities, thus conditioning the results. The training of these professionals should be homogeneous and have a continuous updating programme, as these are issues that evolve very quickly in our society [31].

There are no articles on the evaluation of the results of sex education interventions from a collaborative perspective between educators and health professionals. However, one recent publication has been found which evaluates the impact of the Forma Joven Programme, comparing the participating students with a control group. It concludes that no significant differences are observed in both groups, detecting a deficient systematisation in the implementation of the Programme’s records [32].

When questioned about where they usually get information on sexuality, most adolescents answered that they get it from friends, secondly from talks and workshops at school, and thirdly from the Internet. Although in this study the Internet is not the main source for seeking information on sexuality, according to various publications, a large majority of adolescents use it for this purpose [33].

Adolescents are key stakeholders in sexual health education, yet they are rarely consulted when developing sexual health programmes. Their voices are essential to improving the delivery of relevant and appropriate school-based sexual health education to promote safer adolescent sexual behaviours [34].

A review of the effectiveness of sex education interventions aimed at reducing adolescent sexual risk behaviour demonstrated considerable evidence for interventions that involve parents and the community as participants and that rely on audio-visual media and school-based workshops [35].

In a systematic review carried out by Barriuso-Ortega, Heras-Sevilla and Fernández-Hawrylak (2022) on 16 sexual education programs aimed at adolescents in the school environment in Spain and other countries (USA, China, Uganda), it was found the coexistence of various models of sexual education approach: risk model, abstinence model, biographical-professional model and Comprehensive Sex Education. Of these, the ones that demonstrate the most efficiency in their results are the last two models, which address sexual education in an integral way [36].

Several programs evaluated in Spain have obtained positive results, all of them framed in the biographical-professional model. This is the case of the Agarimos program (Carrera-Fernández et al., 2007) [37], the P.E. Sex program (Claramunt Busó, 2011) [38] and the SOMOS program (Heras Sevilla et al., 2016) [39]. In the current international approach, programs analyzed in the Comprehensive Sex Education model (Chi et al., 2015; Jennings et al., 2014; Rijsdijk et al., 2011) [40–42] can be close to the biographical-professional model, showing greater efficacy in their results compared to those programs that recommend abstinence (Pinkleon et al., 2012) [43]. The authors conclude that sex education should be integrated into the curriculum, offering a comprehensive view of it. Likewise, a rigorous evaluation of all the aspects included in sexual education programs is necessary to demonstrate their effectiveness and improve their contents.

In another systematic review of 60 articles published from different countries on online participatory intervention methods used to promote the sexual health of adolescents and young people, it was found that such interventions are feasible, practical and attractive, although their effectiveness has not been sufficiently evaluated (Philippe Martin et al., 2020) [44].

Regarding the results obtained from the different questions in the “Knowledge and attitudes about sexuality” questionnaire, it should be noted that there is a good level of knowledge about the use of condoms as a method for avoiding the transmission of STIs, although there are still errors and a certain degree of misinformation when it comes to their correct use [45].

Some classic myths about sexuality persist, and in some situations, they can be decisive when it comes to adopting preventive measures to avoid pregnancy risk situations [46]. Furthermore, a considerable percentage of boys and girls continue to focus their sexual practices exclusively on coitus, a reflection of what society values as most important. An important aspect is that stereotypical attitudes persist in relation to sexuality and gender that can even be considered as predisposing to situations of male superiority and violence.

Homosexuality appears as just another sexual orientation option. It is worrying that almost 6% of those surveyed believe that another person can be forced to have sexual relations against their will, and 5% believe that to have a good relationship with a partner, the girl should avoid contradicting the boy. It is essential that these aspects are addressed by the Programme to improve attitudes of gender equality and violence prevention.

During the research process of this study, we have encountered significant difficulties in seeking collaboration from some schools, despite the support of the Health and Education Department and the fact that this is a research project funded and supported by the Ministry of Health and Families of the Andalusian Regional Government.

We consider it important to reflect on this aspect, since the prevailing social “double standard” that demands on the one hand the need to provide sex education content in the school curriculum for young people and on the other hand hinders its application by referring to “possible ethical problems” with parents, does nothing to benefit the “empowerment” of our adolescents to become more autonomous when it comes to making responsible decisions in their lives.

## Conclusions

Affective-sexual education is fundamental in promoting healthy habits amongst adolescents.

Educational and health institutions should facilitate adolescents’ access to quality training in affective-sexual education. To this end, activities targeting adolescents should be promoted, as well as continuous training for the professionals involved.

It would be advisable to improve the system to register the educational activities carried out with young people. Likewise, we recommend the elaboration of materials that facilitate the application of the different contents in the programme to the professionals who work in the counselling centres.

We propose to carry out an in-depth study on the “quality” of educational interventions on sexuality in secondary schools.

## Supplementary Information

**Additional file 1.**

## Data Availability

The datasets generated and analyzed during the current study are not publicly available due to restrictions that applied under the license for the study but are available from the corresponding author AHB on reasonable request.

## Abbreviations

HEI: Higher Education Institute
STI: Sexually Transmitted Infections
