# Supplementary material for: Influence of preventive sex education programmes in compulsory secondary education students: a descriptive observational study
Source: BMC Public Health. 2022 Nov 24;22:2171. doi: 10.1186/s12889-022-14649-w (PMC9700882; doi:10.1186/s12889-022-14649-w)
Supplement: Supplementary file 1 — Additional file 1. [file 12889_2022_14649_MOESM1_ESM.docx]

**Appendices**

**Appendix1.** Parental educational and occupational level

**PARENTAL EDUCATION**

|  | EDUCATIONAL LEVEL | FATHER | MOTHER |
| --- | --- | --- | --- |
| 1 | Cannot read or write |  |  |
| 2 | Can read and write. Incomplete primary studies. |  |  |
| 3 | Complete Primary Studies (EGB, Primary, PCPI). |  |  |
| 4 | Lower Secondary Education (ESO, F.P. Middle Grade) |  |  |
| 5 | Upper Secondary Education (Baccalaureate, BUP, F.P.) |  |  |
| 6 | Medium Grade University Studies (3 courses or Diplomas) |  |  |
| 7 | University Studies Higher Degree (Bachelors, Degrees) |  |  |
| 8 | Doctorate or equivalent (Doctorate, Master's, Postgraduate Studies) |  |  |
| 9 | Other unclassified (specify) |  |  |

**PARENTAL OCCUPATIONAL LEVEL**

|  | CURRENT OCUPPATIONAL LEVEL | FATHER | MOTHER |
| --- | --- | --- | --- |
| 1 | Unemployed. Pensioner. |  |  |
| 2 | Self-employed (entrepreneur): trader, craftsman, street vendor, transport...  Employee: shop assistants, waiters, cooks, mechanics, bricklayers, domestic workers |  |  |
| 3 | Unskilled workers sector 1 (agriculture, farming...): work in the fields, shepherds... |  |  |
| 4 | Skilled workers sector 1 (agriculture, farming...): use machinery and devices... |  |  |
| 5 | Cleric (public and private companies) |  |  |
| 6 | Directors/managers (employers) of establishments with fewer than 10 employees and/or professions linked to diplomas (middle careers): nurses, technical engineers, laboratory technicians, social workers, teachers |  |  |
| 7 | Directors/managers (entrepreneurs) of establishments with more than 10 employees and/or professions linked to degree courses: physicians, veterinarians, architects, senior engineers, economists... |  |  |
| 8 | Other unclassified (specify): |  |  |

Adapted from: Domingo-Salvanya, A., Bacigalupec, A., Carrasco, J.M., Espelt, A., Ferrando, J., Borrell, C., “Propuestas de clase social neoweberiana y neomarxista a partir de la Clasificación Nacional de Ocupaciones 2011 Grupo de Determinantes Sociales de la Sociedad Española de Epidemiología”. Gac. Sanit. 2013;27(3):263–272.

**Appendix 2.** Sexuality knowledge and attitudes questionnaire

- **GENERAL INFO:**

| Gender: | | Age: | | | | | | | | | Academic course: | | | | High School/Town |
| --- | --- | --- | --- | --- | --- | --- | --- | --- | --- | --- | --- | --- | --- | --- | --- |
| Male | Female | 14 | 15 | 16 | 17 | 18 | 19 | 20 | 21 | 22 | 3º ESO | 4º ESO | 1º BACH | 2º BACH |  |
|  |  |  |  |  |  |  |  |  |  |  |  |  |  |  |  |

Where do you usually get information on sexuality? Friends Relatives Internet

Consultancies F. Joven Workshop / Group lectures Other resources: ___________________________ (indicate).

- **QUESTIONNAIRE:**

This questionnaire is anonymous: answer truthfully. Please, before answering, read the sentences carefully and mark with an "X" your degree of agreement or disagreement with them according to the following scale:

**SA:** Strongly Agree **PA**: Partly Agree **PD**: Partly Disagree **SD**: Strongly Disagree

| **Statements to evaluate:** | **Rating:** | | | |
| --- | --- | --- | --- | --- |
|  | **SA** | **PA** | **PD** | **SD** |
| 1. "Girls and boys should talk normally about sexuality." |  |  |  |  |
| 2. "Sexuality is only experienced from adolescence when one becomes sexually mature". |  |  |  |  |
| 3. "Sexuality is limited to the genital organs". |  |  |  |  |
| 4. "The first time a girl has penetrative sex, she cannot get pregnant". |  |  |  |  |
| 5. "If a girl has penetrative sex and does not reach orgasm, she cannot become pregnant". |  |  |  |  |
| 6. "The best way to avoid sexually transmitted diseases is to use condoms in all penetrative relationships". |  |  |  |  |
| 7. "Withdrawal is a very effective method of preventing pregnancy". |  |  |  |  |
| 8. "Condoms are safe if you put them on just before you ejaculate ('coming'). |  |  |  |  |
| 9. "AIDS and other sexually transmitted diseases can be avoided if a condom is used throughout penetrative sex". |  |  |  |  |
| 10. "The most important thing about sex is penetration". |  |  |  |  |
| 11. "Caressing, kissing, mutual masturbation ... can give as much pleasure as making love, but without risk of pregnancy or sexually transmitted diseases". |  |  |  |  |
| 12. "Using a condom allows for safer and equally pleasurable sex". |  |  |  |  |
| 13. "No one can be forced to have sex against their will. |  |  |  |  |
| 14. "Using a condom is not cool because it makes you feel less." |  |  |  |  |
| 15. "If my parents caught me with condoms, they would be very angry with me." |  |  |  |  |
| 16. "Masturbation is a legitimate way to get pleasure. |  |  |  |  |
| 17. "Homosexuality (being gay or lesbian) is a disease. |  |  |  |  |
| 18. "If a boy or girl masturbates, they get pimples". |  |  |  |  |
| 19. "If during your period you have penetrative sex without using a condom, there is no danger of pregnancy". |  |  |  |  |
| 20. "The first time a girl has penetrative sex she always hurts and bleeds". |  |  |  |  |
| 21. "Men and women have the same right to enjoy their sexuality". |  |  |  |  |
| 22. "It is more appropriate for the boy to take the initiative in sexual relations". |  |  |  |  |
| 23. "A certain degree of submission makes girls more attractive". |  |  |  |  |
| 24. "Jealousy is normal when a boy or a girl is in love". |  |  |  |  |
| 25. "To have a good relationship the girl must avoid disagreeing with the boy". |  |  |  |  |
| 26. "Homosexuals (gays, lesbians) should be accepted normally". |  |  |  |  |

Adapted from: <http://www.samfyc.es/Revista/PDF/v3n4/06.pdf>. Medicina de Familia (And) Vol. 3, N.¼ 4, noviembre 2002.

Anuario de Psicología, vol. 34, nº 1 marzo 2003, pp 101-123, Universidad de Barcelona, Facultad de Psicología.

Marchal Torralbo AM, Brado Gagarrido C, Montes Hidalgo J, Tomás Sábado J: Diseño y validación de un instrumento para medir actitudes machistas, violencia y estereotipos en adolescentes. Metas Enferm abril 2018; 21 (3) 11-8.

**Appendix 3.** Professional delivering the "Forma Joven" consultancy

1. NAME OF THE CENTRE (HEI) AND LOCATION:

2. PROFESSIONAL DELIVERING THE “FORMA JOVEN” CONSULTANCY:

- PHYSICIAN
- NURSE
- SOCIAL WORKER
- TEACHER
- COUNSELOR
- OTHER (SPECIFY): ___________________________

3. GENDER:

- MALE
- FEMALE

4. AGE:

5. PREVIOUS TRAINING IN SEX EDUCATION:

- NO
- YES (DETAILS: COURSES, WORKSHOPS, MASTER'S DEGREE, EXPERT ….): ________________________________________

6. EXPERIENCE IN CONSULTANCY (YEARS):

7. OTHER ACTIVITIES OF THE PROGRAM FORMA JOVEN, ABOUT SEXUALITY/CONCEPTION:

- GROUP LECTURES
- WORKSHOPS
- MEDIATOR TRAINING
- WORKSHOPS WITH PARENTS
